# NLRC5 p.Pro191Leu as a genetic susceptibility factor in anti-PIT-1 hypophysitis

**DOI:** 10.1007/s40618-026-02926-z

**Published:** 2026-05-26

**Authors:** Shin Urai, Hironori Bando, Masaaki Yamamoto, Genzo Iguchi, Yutaka Takahashi

**Affiliations:** 1 Division of Diabetes and Endocrinology, Department of Internal Medicine, Kobe University Graduate School of Medicine, 7-5-1 Kusunoki-cho, Chuo-ku, 650-0017 Kobe, Hyogo Japan; 2 Department of Diabetes and Endocrinology, Nara Medical University, Kashihara, Japan; 3 Faculty of Clinical Nutrition and Dietetics, Department of Clinical Nutrition and Dietetics, Konan Women’s University, Kobe, Japan

**Keywords:** NLRC5, PIT-1, Autoimmune hypophysitis, Paraneoplastic syndrome

## Abstract

**Purpose:**

Anti-PIT-1 hypophysitis is a rare T cell-mediated autoimmune pituitary disorder presenting as a paraneoplastic syndrome or after immune checkpoint inhibitor (ICI) therapy, and its predominance in Japan suggests an underlying genetic predisposition. The NLR family CARD domain containing 5 (*NLRC5*) encodes a key transcriptional regulator of major histocompatibility complex (MHC) class I, and its p.Pro191Leu missense variant is associated with ICI-related endocrinopathies and enhanced interferon-γ responses. Therefore, we examined whether NLRC5 p.Pro191Leu is enriched in anti-PIT-1 hypophysitis.

**Methods:**

We genotyped the NLRC5 p.Pro191Leu variant (c.572 C > T) in seven Japanese individuals with anti-PIT-1 hypophysitis for whom genomic DNA was available. The carrier frequency observed in this cohort was compared with ancestry-matched expectations from the Tohoku Medical Megabank 61KJPN Japanese reference panel using a prespecified one-sided exact binomial test. Sensitivity analyses included Mid-*P* adjustment, risk differences, and risk ratios.

**Results:**

Among seven genotyped Japanese individuals with anti-PIT-1 hypophysitis, five (71.4%) were carriers, exceeding the expected ancestry-matched frequency from the Japanese reference panel (expected carrier frequency 37.7%; exact *p* = 0.076; mid-*P* = 0.045). The effect sizes were risk difference + 0.337 and risk ratio 1.89.

**Conclusion:**

This study provides suggestive evidence that the NLRC5 p.Pro191Leu variant may confer genetic susceptibility to anti-PIT-1 hypophysitis in the Japanese population, based on its enrichment relative to ancestry-matched reference panels. These findings, together with NLRC5’s role as the master regulator of MHC class I expression, propose a novel and testable pathogenic model.

**Supplementary Information:**

The online version contains supplementary material available at 10.1007/s40618-026-02926-z.

## Introduction

Anti-pituitary transcription factor-1 (PIT-1) hypophysitis is a distinct T cell-mediated autoimmune disorder recognized as a novel acquired cause of hypopituitarism [1–3]. It represents a form of paraneoplastic autoimmune hypophysitis driven by human leukocyte antigen (HLA)-restricted immune responses against PIT-1-expressing cells. This condition is characterized by selectively impaired secretion of PIT-1-related anterior pituitary hormones, including growth hormone, prolactin, and thyroid-stimulating hormone, resulting in hypopituitarism with a distinctive hormonal profile [1, 3]. Although the precise genetic determinants remain unknown, all reported cases have been concentrated in Japan, suggesting ancestry-related susceptibility.

The growing number of cases in the immune checkpoint inhibitor (ICI) era has renewed interest in the immunogenetic architecture of anti-PIT-1 hypophysitis, which, although it is primarily a paraneoplastic syndrome, can also emerge in an ICI-related form after treatment for underlying malignancies [4]. Recently, ICI-induced diabetes has been linked to germline variation in NLR family CARD domain containing 5 (*NLRC5*), specifically the enriched missense variant p.Pro191Leu, suggesting context-dependent modulation of antigen-processing machinery [5, 6]. Small case series of patients with ICI-related hypophysitis have similarly suggested an enrichment signal of NLRC5 p.Pro191Leu [6]. However, it remains unclear whether NLRC5 p.Pro191Leu also confers susceptibility to paraneoplastic autoimmune hypophysitis, including anti-PIT-1 hypophysitis, which occurs in a related but distinct clinical setting.

NLRC5 is the master transcriptional activator of the major histocompatibility complex (MHC) class I pathway, corresponding to HLA class I in humans, and antigen-processing and presentation machinery, enabling antigen presentation to cluster of differentiation (CD) 8^+^ T cells [7–9]. Based on this mechanistic and epidemiologic background, we focused on NLRC5 p.Pro191Leu as a probable susceptibility variant for evaluation in anti-PIT-1 hypophysitis. Our objective was to assess whether carrier status is enriched in a Japanese case series relative to ancestry-matched population expectations and to characterize any observed signals using orthogonal resources. This report extends prior observations of NLRC5 p.Pro191Leu enrichment in ICI-related endocrinopathies to include autoimmune hypophysitis.

## Materials and methods

The diagnosis of anti-PIT-1 hypophysitis was established based on previously reported diagnostic criteria, integrating clinical and serologic findings together with an appropriate neoplastic background, with pathologic confirmation where available. Clinical characteristics of the individuals, including detailed endocrine profiles, imaging findings, and clinical course, have been detailed previously [1, 4, 10]. Among 10 Japanese individuals with anti-PIT-1 hypophysitis from our prior reports [1, 10], we analyzed peripheral blood DNA from seven (cases #1, #3, #5, #6, #8–10), constituting the present genetic analysis set.

We prespecified NLRC5 p.Pro191Leu (GRCh38, chr16:57025515 C > T; c.572 C > T; rs74439742) as a susceptibility variant and genotyped it by Sanger sequencing of whole-blood genomic DNA using a previously published primer [6]. Expected carrier frequencies were derived under Hardy–Weinberg equilibrium (HWE) from the Tohoku Medical Megabank (ToMMo) 61KJPN Japanese reference panel, with other Japanese and global datasets used descriptively [11–13]. The observed carrier frequency in anti-PIT-1 hypophysitis was compared with the ToMMo expectation using a prespecified one-sided exact binomial test; a one-sided mid-*P* value, risk difference, risk ratio, and an odds ratio versus ToMMo counts were calculated as additional metrics. An allele-based analysis was also performed as a supportive measure. Standard public resources, including GTEx for *NLRC5* tissue expression [14], in silico prediction tools for functional prediction of p.Pro191Leu [15–20], eQTLGen for whole-blood cis/trans expression quantitative trait loci (eQTL) [21], and LDmatrix for linkage disequilibrium at the *NLRC5* locus [22], were used to characterize expression, functional impact, and regulatory context. Full protocols, reference panel selection, and analytic pipelines are provided in the **Supplementary Methods**.

## Results

### Genetic findings in anti-PIT-1 hypophysitis, population reference, and statistical comparison

**Fig. 1:**
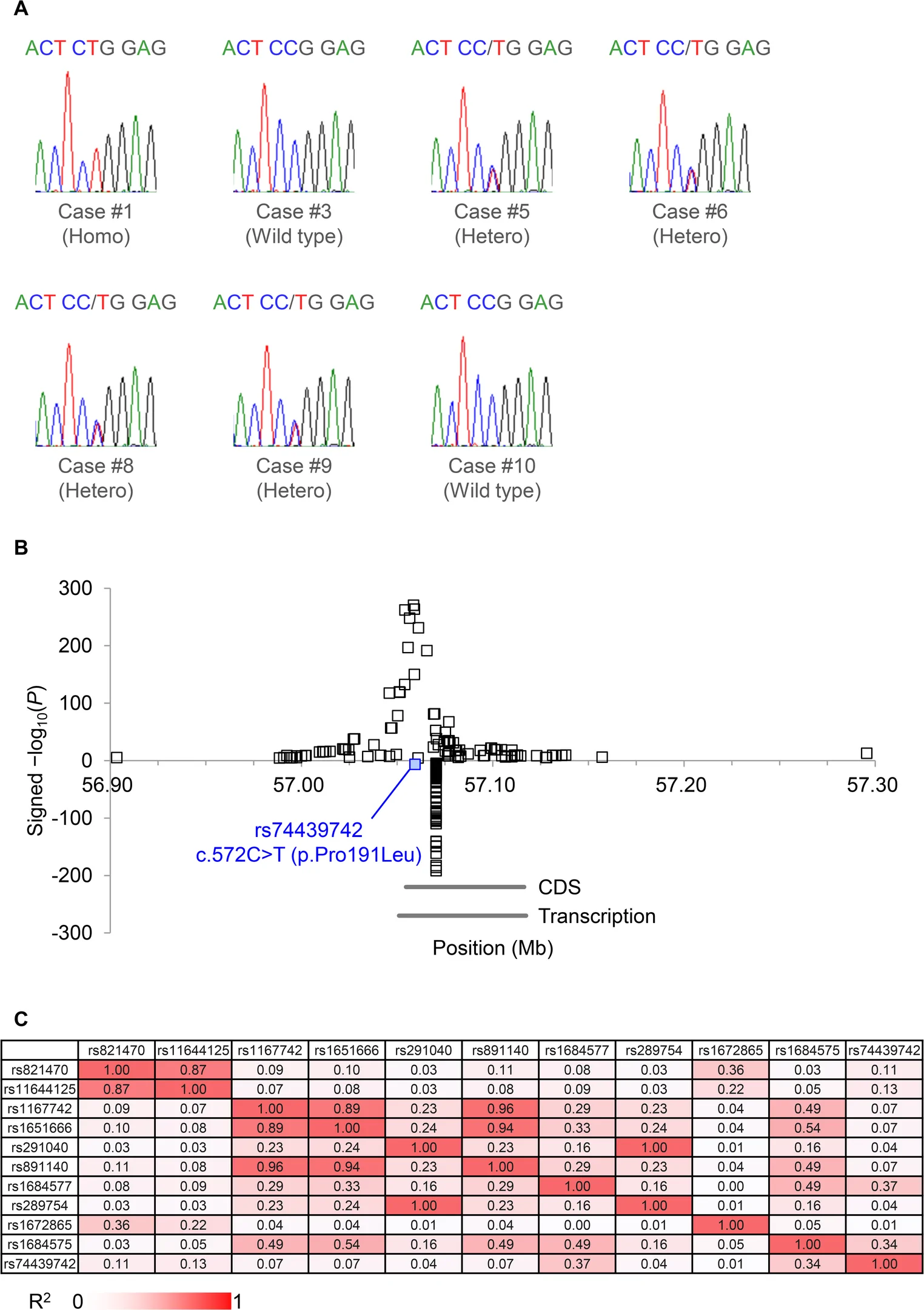
Genetic and genomic characterization of the NLRC5 p.Pro191Leu variant identified in individuals with anti-PIT-1 hypophysitis. (**A**) Representative Sanger sequencing chromatograms of *NLRC5* exon 6 showing the c.572 C > T substitution (p.Pro191Leu). Case #1 is homozygous for the variant (TT; Leu/Leu), Cases #5, #6, #8, and #9 are heterozygous (CT; Pro/Leu), and Cases #3 and #10 are wild type (CC; Pro/Pro). The codon corresponding to residue 191 (CCG/CTG) is indicated above each trace. (**B**) cis-eQTL signals across the *NLRC5* locus plotted using the GRCh38 genomic position. Only variants with statistically significant cis-eQTL *p*-values are shown. Plots are separated by the Z-score sign, and the y-axis represents the signed − log₁₀(*P*) (positive for positive Z, negative for negative Z); the x-axis indicates genomic position (Mb, GRCh38). Coding sequence (CDS) tracks and transcription are shown at the bottom. (**C**) Pairwise linkage disequilibrium (R²) between rs74439742 and the top 10 *NLRC5* cis-eQTL SNPs from the Japanese reference panel. The matrix lists the R² values for each SNP pair, with higher values indicating stronger linkage disequilibrium. Across these signals, rs74439742 shows generally low-to-moderate linkage disequilibrium with the top cis-eQTL variants

All individuals included in this study displayed hypopituitarism characterized by deficiencies in PIT-1-related hormones. No individual had arginine vasopressin deficiency. Two of the seven individuals had a history of ICI therapy (anti-PD-1 agents), with initial reductions in thyroid-stimulating hormone levels observed within 3–4 weeks after treatment initiation, followed by reduction in growth hormone and prolactin levels, leading to the diagnosis of anti-PIT-1 hypophysitis (cases #9 and #10). In anti-PIT-1 hypophysitis, among seven individuals with available genomic DNA, the NLRC5 p.Pro191Leu (GRCh38, chr16:57025515 C > T; c.572 C > T; rs74439742) distribution comprised one homozygous-alternate and four heterozygous carriers versus two non-carriers (5/7; 71.4%) (Fig. 1A; Table 1). The underlying oncologic diagnoses included three thymomas (cases #1, #3, and #8) and four non-thymoma malignancies (cases #5, #6, #9–10).

**Table 1:** Allele frequency and carrier frequency for *NLRC5* rs74439742 (p.Pro191Leu)

Source / database	Cohort / dataset	Population/ancestry	*N* (individuals)	AF (alternate allele)	Carrier frequency (%)^a^
This study	Anti-PIT-1 hypophysitis cohort	Japanese	7	—^b^	71.4 (5/7)
Japanese reference databases					
jMorp (ToMMo)	61KJPN	Japanese	61,242	0.211	37.7
jMorp (ToMMo)	60KJPN	Japanese	59,940	0.210	37.6
jMorp (ToMMo)	54KJPN	Japanese	54,302	0.210	37.6
jMorp (ToMMo)	38KJPN	Japanese	38,722	0.211	37.7
jMorp (ToMMo)	14KJPN	Japanese	14,129	0.208	37.2
TogoVar	GEM-J WGA	Japanese	—	0.202	36.3
TogoVar	JGA-WES	Japanese	—	0.244	42.8
Global reference databases					
gnomAD v4	Genomes	Global (multi-ancestry)	—	0.132	24.6
TogoVar	JGA-WGS	Global/Total	—	0.199	35.8
TogoVar	NCBN	Global/Total	—	0.183	33.3

Allele and carrier frequencies for *NLRC5* rs74439742 (p.Pro191Leu) are shown

^a^ Carrier frequency (≥ 1 alternate allele) for population datasets was derived under the Hardy–Weinberg equilibrium as 1 − (1 − AF)^2^

^b^ AF was not used as the primary measure for the present case-enriched cohort; the observed carrier proportion is shown here instead

In the ancestry-matched Japanese ToMMo 61KJPN panel (*n* = 61,242), the alternate allele frequency (AF) was 0.211, corresponding under HWE to a carrier frequency of 0.377. Across multiple Japanese reference panels, the AF was consistently ≈ 0.21 (implied carrier frequency ≈ 0.38), whereas global multi-ancestry panels showed a lower AF (≈ 0.13), indicating a higher background frequency in Japanese than in worldwide populations (Table 1).

Relative to the Japanese reference carrier frequency of 0.377, the observed 71.4% carrier proportion in anti-PIT-1 hypophysitis (two-sided exact 95% CI 0.29–0.96) yielded *p* = 0.076 on the one-sided exact binomial test. Notably, a sensitivity analysis using the mid-*P* approach, which is less conservative for small sample sizes, resulted in a mid-*P* value of 0.045. The effect sizes were a risk difference of + 0.337 and risk ratio of 1.89. As additional context, the 2 × 2 comparison with ToMMo counts produced an odds ratio of 4.12 (95% CI 0.80–21.3), suggesting a large point estimate with wide uncertainty attributable to the small case denominator rather than population count instability. These statistics indicated a directional enrichment signal compatible with an increased carrier frequency in anti-PIT-1 hypophysitis yet remains exploratory.

Consistent with the prespecified analytic framework, allele-based analysis was performed as a supportive analysis. The results showed 6 alternate alleles among 14 alleles in the anti-PIT-1 hypophysitis cohort (allele frequency 0.429), compared with an expected allele frequency of 0.211 in the ToMMo 61KJPN Japanese reference panel. A prespecified one-sided exact binomial test yielded an exact *p*-value of 0.055 and a one-sided mid-*P* value of 0.035. The corresponding effect sizes were a risk difference of + 0.218 and risk ratio of 2.03.

### Functional annotations for NLRC5 p.Pro191Leu

In bulk RNA-seq datasets (GTEx v10), *NLRC5* was highly expressed in lymphoid and blood compartments, with median transcripts per million (TPM) of 68.50 in spleen, 52.25 in EBV-transformed lymphocytes (immortalized), and 41.73 in whole blood, and was also appreciably expressed in the pituitary (TPM 18.50), supporting tissue-intrinsic plausibility for an NLRC5-linked mechanism in PIT-1 autoimmunity.

Across algorithms, in silico predictions for NLRC5 p.Pro191Leu were predominantly benign: PolyPhen-2 = 0.00 (benign), SIFT = 0.05 (borderline tolerated), and MutationTaster = Benign, whereas Align-GVGD = Class C65 (compatible with a context-dependent functional effect). Splice AI predicted no splice impact (Δ score = 0.01) and CADD was low (PHRED = 6.136).

In eQTLGen (steady-state, non-stimulated bulk whole blood), rs74439742 (GRCh38, chr16:57025515 C > T; c.572 C > T; p.Pro191Leu) acted as a cis-eQTL for *NLRC5* with a negative effect direction (Z = − 5.14; *p* = 2.79 × 10^− 7^; FDR = 8.65 × 10^− 4^; *n* = 13,100), indicating lower basal *NLRC5* expression for the assessed allele. In contrast, pituitary-specific eQTL statistics for this variant were unavailable in public resources (GTEx), precluding tissue-matched eQTL inference. At the locus level, rs74439742 was also associated with the neighboring noncoding transcript (RP11-322D14.1; Z = − 6.80; *p* = 1.05 × 10^− 11^), consistent with regional regulatory activity. Regional inspection of the *NLRC5* locus revealed a principal cis-eQTL cluster spanning upstream/regulatory and intronic regions, with rs74439742 mapped near the center of this cluster (Fig. 1B). In a Japanese reference panel (LDmatrix), rs74439742 showed an overall low linkage disequilibrium with lead cis-regulatory variants (Fig. 1C). A prespecified catalog search in eQTLGen did not identify study-wide significant trans-eQTLs attributable to rs74439742 in steady-state whole blood. Together with the negative cis direction at rest, these findings indicated that any downstream coordination across the antigen-processing pathway is undetectable at baseline.

## Discussion

In this study, carriers of NLRC5 p.Pro191Leu constituted 71.4% (5/7) of the genetic analysis set, exceeding ancestry-matched expectations from Japanese population panels. Although the prespecified exact test was borderline (*p* = 0.076) owing to small sample size, the complementary mid-*P* value supported the same directional enrichment signal (mid-*P* = 0.045). These results provided suggestive evidence of a genetic susceptibility effect and supported the hypothesis that this variant contributes to disease risk. This observation is notable considering the relatively high background frequency of NLRC5 p.Pro191Leu in the Japanese reference population and the predominance of published anti-PIT-1 hypophysitis cases in Japan. This geographic pattern may reflect an ancestry-related immunogenetic structure, differential recognition and reporting, or both, and cannot be resolved in this small series, highlighting the need for larger epidemiological studies.

Mechanistically, public datasets indicate that the variant functions as a cis-eQTL for *NLRC5* associated with lower basal whole blood expression. This finding is consistent with subtle regulatory differences at rest; however, the pathogenic relevance of the variant likely manifests under inflammatory conditions. Notably, prior functional analysis in ICI-treated individuals demonstrated that NLRC5 p.Pro191Leu heightens interferon-γ–induced upregulation of antigen-processing machinery transcripts [6]. These biological data support a pathogenic role in anti-PIT-1 hypophysitis and suggest that the enrichment signal reflects more than a small-sample artifact, raising the possibility of a shared mechanism with ICI-induced autoimmunity involving hyperinducible antigen presentation under inflammatory stress. These findings, combined with the established functional impact on MHC class I regulation [9, 23], support NLRC5 p.Pro191Leu as a plausible susceptibility factor for developing anti-PIT-1 hypophysitis.

Several limitations tempered these inferences. First, all comparisons relied on external population references in the absence of disease-matched control groups (e.g., individuals with other pituitary or thymic diseases without anti-PIT-1 hypophysitis), which prevented determining whether the observed enrichment reflected susceptibility specific to anti-PIT-1 hypophysitis. Second, statistical statements should be regarded as enrichment signals rather than definitive evidence, given wide CIs and a primary *p*-value that did not reach significance using the conservative exact test. Third, pituitary-specific eQTL statistics for rs74439742 are not available in public resources, precluding tissue-matched validation and necessitating the use of whole-blood resting cis signals as directional rather than tissue-definitive evidence. Finally, computational annotations and bulk eQTLs provide supportive and non-definitive information and were used to propose a plausible, testable mechanistic model rather than to assert causality.

As many individuals with anti-PIT-1 hypophysitis are undergoing oncologic treatment, obtaining sufficient peripheral blood mononuclear cells is often difficult. Accordingly, individual-derived induced pluripotent stem cell platforms offer a practical, disease-proximal alternative for genotype-stratified activation-response testing [2]. Such studies are needed to determine whether interferon-conditioned shifts in NLRC5-regulated antigen processing in circulating or tissue-resident immune cells enhance CD8⁺ T cell recognition and cytotoxicity. They may also help clarify whether the observed geographic clustering reflects ancestry-linked susceptibility or differential ascertainment, thereby informing subsequent mechanistic and epidemiological studies.

## Electronic Supplementary Material

Below is the link to the electronic supplementary material.

Supplementary Material 1

## Data Availability

All relevant data are presented in the manuscript and information files.
