# Supplementary material for: NLRC5 p.Pro191Leu as a genetic susceptibility factor in anti-PIT-1 hypophysitis
Source: J Endocrinol Invest. 2026 May 26;49(9):2571–6. doi: 10.1007/s40618-026-02926-z (PMC13498477; doi:10.1007/s40618-026-02926-z)
Supplement: Supplementary file 1 [file 40618_2026_2926_MOESM1_ESM.pdf]

Supplementary Methods

Study design, participants, and genotyping

We analyzed individuals with anti-PIT-1 hypophysitis identified in our previous reports, noting that 10 Japanese individuals were previously described by our group [1, 10]. These publications provided the clinical and immunological characterization of this cohort but did not investigate the germline genetic association examined in the present study. For the current analysis, genomic DNA was available for seven individuals, who constituted the genetic analysis set (cases #1, #3, #5, #6, and #8–10). The diagnosis of anti-PIT-1 hypophysitis was established using prespecified criteria integrating selective deficiencies of PIT-1-related anterior pituitary hormones (growth hormone, prolactin, and thyroid-stimulating hormone) with serologic evidence, including anti-PIT-1 antibodies and/or PIT-1-reactive T cells [1, 3]. An underlying neoplastic lesion was considered supportive of the diagnosis and pathogenesis but was not required to be clinically apparent when endocrine abnormalities were first recognized.

Genomic DNA was extracted from peripheral whole blood. The *NLRC5* exon containing p.Pro191Leu (GRCh38, chr16:57025515C>T; c.572C>T; rs74439742) was amplified via polymerase chain reaction using previously published primer pairs and sequenced with Sanger sequencing [6]. Each individual with available genomic DNA was genotyped for NLRC5 p.Pro191Leu and categorized as homozygous-alternate, heterozygous, or non-carrier. All procedures adhered to institutional ethics oversight, and each individual provided written informed consent permitting journal submission and publication.

Prespecified population reference and primary measure of association

The primary association measure was the carrier frequency (heterozygous or homozygous-alternate) of NLRC5 p.Pro191Leu (GRCh38, chr16:57025515C>T; c.572C>T; rs74439742) in this cohort. The Japanese background alternate allele frequency (AF) was defined using population-based whole-genome panels from the Tohoku Medical Megabank (ToMMo) accessed via the jMorp portal [11]. The 61KJPN panel served as the primary Japanese reference. The 60KJPN, 54KJPN, 38KJPN, and 14KJPN panels were used to verify the internal consistency of the Japanese reference. For hypothesis testing in the genetic analysis set, the baseline carrier frequency (heterozygous + homozygous-alternate) was derived from the AF under the Hardy–Weinberg

equilibrium (HWE) as Pr(carrier) = $1 - (1 - AF)^2$. To cross-validate Japanese AF estimates, TogoVar was queried and retrieved for dataset-specific AFs for rs74439742 from GEM-J WGA and JGA-WES (Japanese cohorts) [12]. For global, non-Japanese context, gnomAD v4 Genomes (Total) was referenced, with JGA-WGS and NCBN serving only as concordance checks [12, 13]. These population resources were used solely to derive ancestry-matched background frequencies.

Carrier frequency was summarized with two-sided 95% Clopper–Pearson exact confidence intervals (CIs). The primary analysis evaluated whether the observed carrier frequency in the anti-PIT-1 hypophysitis exceeded the ancestry-matched reference carrier frequency derived from ToMMo/jMorp under the HWE assumption using a one-sided exact binomial test (alpha-value = 0.05). As the exact test can be conservative in small samples owing to binomial distribution discreteness, a one-sided mid-*P* value was additionally reported as a sensitivity analysis. Effect sizes were expressed as risk differences and risk ratios relative to the reference. As contextual analysis, a 2×2 comparison using general population carrier counts was constructed to obtain an odds ratio with a 95% CI.

As a supportive analysis, allele frequency was compared with that in the ancestry-matched ToMMo 61KJPN Japanese reference panel using a one-sided exact binomial test while treating the observed number of alternate alleles as binomial counts under the reference allele frequency. A one-sided mid-*P* value, risk difference, and risk ratio were calculated as sensitivity measures.

Exact/binomial procedures were performed using IBM SPSS Statistics v29 (IBM Corporation, Armonk, NY, USA), and the mid-*P* value was computed using Microsoft Excel.

Functional annotation: in silico prediction, expression profiling, and linkage disequilibrium

Tissue-level *NLRC5* expression was summarized from bulk datasets (GTEx v10), focusing on whole blood to align with available eQTL resources and the pituitary gland to establish organ-level relevance to PIT-1 autoimmunity [14]. These profiles were used descriptively to frame regulatory analyses; no differential expression testing was performed in this study. To assess potential functional consequences of rs74439742 (GRCh38, chr16:57025515C>T; c.572C>T; p.Pro191Leu), we compiled *in silico* annotations from Polymorphism Phenotyping v2 (PolyPhen-2), Sorting Intolerant From Tolerated (SIFT), MutationTaster,

Align–Grantham Variation Grantham Deviation (Align-GVGD) (amino-acid substitution impact), Splice AI (splice-effect prediction), and Combined Annotation Dependent Depletion (CADD) (integrated deleteriousness score) [15–20]. For regulatory evidence, cis-expression quantitative trait loci (eQTL) directionality for rs74439742 was queried in eQTLGen using steady-state (non-stimulated) bulk whole-blood data [21]. We also prespecified a catalog search for trans-eQTL signals for rs74439742 in eQTLGen, with any findings (or their absence) reported descriptively in the Results without hypothesis testing. Linkage disequilibrium between rs74439742 and the top 10 *NLRC5* cis-eQTL variants (by absolute Z-score) was evaluated as R^2 using a Japanese reference panel via LDmatrix to assess independence from dominant regulatory haplotypes [22].
